# Evaluation of Pesticides on Detritus-Inhabiting and Root-Associated Fungi in Aquatic Habitats and Potential Implications

**DOI:** 10.3390/d16050255

**Published:** 2024-04-24

**Authors:** Daniel B. Raudabaugh, Andrew N. Miller, Claudia K. Gunsch

**Affiliations:** 1 Department of Civil and Environmental Engineering, Duke University, Durham, NC 27708, USA; 2 Department of Botany and Plant Pathology, Purdue University, West Lafayette, IN 47907, USA; 3 Illinois Natural History Survey, University of Illinois at Urbana-Champaign, Champaign, IL 61801, USA

**Keywords:** atrazine, cypermethrin, malathion, mancozeb, cellulase, starch degradation, tannase

## Abstract

Pesticide contamination of aquatic ecosystems poses a significant threat to humans and can adversely affect fungal-driven processes in these understudied habitats. Here, we investigated the effects of four pesticides on detritus-inhabiting and plant root-associated fungi from streams, peatlands, and saltwater marshes. Additionally, we assessed the isolates’ capacities to degrade three carbon sources to understand the impact of pesticides on fungal-driven processes. Pesticide assays were conducted in 96-well glass-coated plates, with fungal growth measured using a UV-Vis spectrophotometer set to 595 nm. Assays included technical replication (n = 6), replication over time (n = 2), negative controls, and carry-over controls. In total, we assayed more than 153 isolates, representing up to 97 fungal genera. Results showed that 1.9%, 49.7%, 3.1%, and 5.6% of the isolates exhibited consistently lower growth when exposed to atrazine, mancozeb, cypermethrin, and malathion, respectively. Furthermore, 101 isolates, comprising 87 genera, were tested for cellulase, starch degradation, and tannase activity, with 41.6%, 28.7%, and 30.7% of the isolates testing positive, respectively. These findings suggest that while many species demonstrate functional redundancy, some fungal species are sensitive to current environmental pesticide levels, which affects their growth and may have broader implications on ecosystem health.

## 1. Introduction

Out of the estimated 2.5 million fungal species worldwide [1], around 3870 species have been identified in freshwater habitats [2]. These habitats include lentic environments like lakes, ponds, and wetlands, as well as lotic systems such as streams and rivers [3]. The fungal species found in freshwater ecosystems comprise representatives from the Ascomycota, Chytridiomycota, and, to a lesser extent, Basidiomycota [4]. Moreover, recent studies have revealed a diverse array of fungi associated with the roots of submerged aquatic plants [5,6]. Within the marine environment, there are at least 200 obligatory marine fungi within mangrove forests [7], and at least 465 filamentous fungi have been reported from marine environments as of 2003 [4]. The importance of fungi as key drivers of ecosystem functions and their symbiotic relationships with host plants in aquatic environments has been extensively researched [8–12].

Aquatic fungi are pivotal in the degradation of submerged dead plant matter [3]. Within plant matter, fungi can quickly utilize simple compounds (amino acids, starch, and sugars), while the breakdown of the three main structural components (cellulose, hemi-cellulose, and lignin) occurs over a longer period [13]. The smaller refractory substances (phenols and tannins) are also known to be broken down or modified by aquatic fungi [14]. Aquatic fungi are also important food sources for aquatic shredders and zooplankton, and they shape the seasonal succession of phytoplankton [11,15]. Regarding host plants, dark septate root-associated endophytic fungi have been shown to improve host tolerance to drought, heavy-metal contamination, and salt stress [16–18]. Unfortunately, in 2007, it was reported [19] that most U.S. streams in agricultural and urban settings contained at least one detectable pesticide and that 31–83% of streams and stream sediments contained levels of pesticides greater than aquatic-life benchmarks.

Globally, 10% of pesticides in use are fungicides, 40% are herbicides, and 17% are insecticides [20]. The remaining 33% are used to limit a variety of pests, including rodenticides, bactericides, and larvicides. China, the United States of America, and Argentina are the top consumers of pesticides, using around 2.4 million kilograms combined annually.

In 2015, research on the effects of pesticides on non-targeted organisms suggested that, in general, pesticides of all types are detrimental to fungal species [21]. In soils, it has been shown that insecticides and herbicides can inhibit common soil fungi [22,23]. A recent study [24] concluded that the fungicide thifluzamide (10.0 mg/kg) could decrease both soil fungal diversity and abundance, which altered the original fungal-driven processes. Presently, a significant knowledge gap exists in connecting fungal species-level contributions to crucial ecological processes driven by fungi in aquatic ecosystems [25].

Elucidating environmental function and be completed in several ways, one of which is assaying bulk microbial function or via isolating and assaying individual species to determine their ecological function. Some researchers who use ecological community-based analyses suggest that similar species provide functional redundancy [26], while other studies highlight specific species [27] as critical for stable community processes, especially in the carbon and nitrogen cycles. Additionally, there is evidence suggesting that the overall extent of ecological processes results from species complementing each other functionally [26]. This underscores the utility of bulk community functional analyses in outlining the range of functions a community provides. However, unlike individual species’ functional analyses, they fall short in assessing functional redundancy among species or variations in functional significance between species. Functional redundancy refers to a species’ capacity to degrade the same substrate, while functional significance pertains to how effectively a species can degrade a substrate, gauged by measuring enzymatic activity.

Due to current levels of pesticide use, the development of new pesticides, and the high incidence of aquatic contamination, it is important to understand how these chemicals affect fungal-driven environmental processes within these ecosystems. Therefore, the research objectives of this work were to (1) conduct toxicity assays, using four widely used pesticides, on a broad taxonomic range of detritus inhabiting and root-associated fungi from peatland, stream, and marsh habitats, (2) assay isolated fungi to determine their ability to degrade common organic substrates—cellulose, starch, and tannin—and (3) link pesticide sensitivity to ecological processes to provide greater understanding of how pesticides may effect degradation in aquatic ecosystems.

## 2. Materials and Methods

### 2.1. Sampling Location and Fungal Isolation

Freshwater detritus inhabiting fungi were isolated from peatlands and streams in Pennsylvania and Wisconsin [28] (Figure S1, Table 1). In addition, root-associated fungi were isolated from two common saltwater marsh plants, *Phragmites australis* and *Spartina alterniflora*, located in the Chesapeake Bay region, Newport News, VA, USA (Figure S1, Table 1).

The isolation of detritus-inhabiting fungi was conducted following methods described in [28]. In brief, fine detritus was collected from each location and transported to the lab at 4 °C. Two grams of each sample were blended in 200 mL distilled water for 15 s, and 300 μL of this mixture was lawn-plated on three different media: malt extract agar (Difco, Franklin Lakes, NJ, USA) supplemented with 0.1 g/L chloramphenicol, Rose Bengal agar, and tea leaf agar [29]. Plates were sealed with Parafilm and incubated for 24 h in the dark. Spring (May) and Winter (November) samples were incubated for two weeks at 7 °C, followed by two weeks at 14 °C and 21 °C. Summer (August) samples were incubated for two weeks at 14 °C and 21 °C. Plates were monitored, and fungal colonies were isolated until axenic cultures were obtained.

For root-associated fungal endophytes, plant specimens were collected in November 2019, placed in individual sterile bags, stored on ice in a cooler, and transported to the lab for processing. The top portion of each plant was removed prior to root cleaning. Roots were thoroughly cleaned with tap water to remove visible debris and surface sterilized according to [30], dried for 5 min. On autoclaved tissue paper in a laminar flow hood, cut into 5 cm pieces using a sterile razor blade, and plated onto cycloheximide nitrogen-free medium [31], malt extract agar (Difco), and Spezieller Nährstoffarmer agar [32]. Isolates were incubated under 24-h darkness at a temperature of 18–22 °C. Fungal colonies growing out of the sterilized tissues were transferred to malt extract agar plates, wrapped with Parafilm, and allowed to grow before molecular identification and toxicity assays. Roots were cut after sterilization to prevent sterilization fluid from entering the root tissue, which could result in over-sterilization. All isolates utilized within this experiment are found below (Table 2) and within the Supplemental Materials (Data S1).

### 2.2. Molecular Identification of Fungal Isolates

Fungal samples were identified by analyzing the nuclear ribosomal ITS region (ITS1–5.8s-ITS2). The extraction of fungal DNA followed a modified NaOH-based procedure [33], detailed in [28]. In summary, fresh mycelium was pulverized within a 1.5 mL centrifuge tube containing 200 μL of 0.5 M NaOH solution, then centrifuged at 16,873× *g* for 2 min. Next, 5 μL of the supernatant was mixed with 495 μL of 100 mM Tris-HCl buffer (pH 8.5–8.9). PCR was performed using a Bio-Rad PTC 200 thermal cycler with a 25 μL total reaction volume (12.5 μL GoTaq^®^ Green Master Mix, 1 μL of each 10 μM primer ITS1F (5′-CTTGGTCATTTAGAGGAAGTAA-3′) and ITS4 (5′-TCCTCCGCTTATTGATATGC-3′) [34,35], 3 μL Tris-HCl-DNA extraction solution, and 7.5 μL DNA-free water). Gel electrophoresis using a 1% agarose gel stained with ethidium bromide confirmed the presence of PCR products before purification with a Wizard^®^ SV Gel and PCR Clean-Up System (Promega, Madison, WI, USA). Sequencing of the ITS region was performed in one direction using the ITS5 primer (5′-GGAAGTAAAAGTCGTAACAAGG-3′) [35]. Sequencing was completed using a BigDye^®^ Terminator 3.1 cycle sequencing kit (Applied Biosystems, Waltham, MA, USA) on an Applied Biosystems 3730 XL high-throughput capillary sequencer. Sequence analysis and editing were carried out using Sequencher^®^ 5.4.6 DNA sequence analysis software (Gene Codes Corporation, Ann Arbor, MI, USA), and all sequences were compared against the NCBI database using BLASTn analysis [36].

### 2.3. Pesticides

Pesticide selection was made to include different modes of action and included the most common pesticides in the current and future global usage rankings [37]. The pesticides used in this study were as follows: (1) Mancozeb, a dithiocarbamate (Biosynth, Louisville, KY, USA); (2) malathion, an organophosphate (Toronto Research Chemicals, Toronto, ON, Canada); (3) cypermethrin, a pyrethroid (Toronto Research Chemicals); (4) atrazine, a triazine (TCI America, Portland, OR, USA). Dithiocarbamates are bactericides and fungicides that inhibit metal-dependent and sulfhydryl enzyme systems. Two groups of insecticides were evaluated: organophosphates, which inhibit acetylcholinesterase in the nervous system, and pyrethroids, which target nerve sodium channels. Finally, triazines are herbicides that inhibit photosynthesis in plants. Specifically, atrazine has been shown to be persistent in surface water [38] and is a common environmental contaminant.

#### Toxicity Assays—Detritus Inhabiting and Root-Associated Fungi

The effects of pesticide exposure on fungal growth were assessed using a microplate reader because culture tissue absorbance has been shown to correlate with dry fungal weight regardless of inoculum type [39]. We evaluated between 153–162 fungal isolates consisting of up to 97 genera. All assays were performed in 370 μL 96-well glass-coated 7 mm RDFlat base plates (Chrom Tech^®^ Inc., Apple Valley, MN, USA) covered with a Breathe-Easy^®^ sealing membrane (Sigma-Aldrich, St. Louis, MO, USA) to prevent contamination. Each pesticide was dissolved in methanol, added to each well at the targeted concentration (Table 3), placed under a laminar flow hood until the solvent evaporated, and inoculated with 250 μL filtered potato dextrose broth containing 3 × 10^3^ ± 3 × 10^2^ conidia/hyphal fragments or yeast cells per μL. Each plate included 8 negative controls with no pesticide, and 6 technical replicates were completed for each targeted pesticide concentration. All plates were covered with a breathable sealing membrane and placed under 24-h darkness at 21 °C. Assays were evaluated after 3 days of exposure by measuring absorbance at 595 nm using a Tecan Infinite M1000 Pro with Magellan v 7.2 software. Assays were repeated twice over time, and carry-over controls were completed for 24 fungi, with 8 technical replicates per isolate, to verify there was no pesticide carry-over when changing from one pesticide to another. To determine the influence of each pesticide on fungal growth, we used a conservative limit of detection equal to mean ±3 standard deviation of each pesticide control group [40] as compared to the more stringent mean ±2 standard deviation to account for only 2 replications over time. Therefore, the absorbance assays were scored as follows: (1) absorbance within the limit of detections was equal to non-exposure, (2) isolate was considered to have positive growth if the absorbance was above the limit of detection, and (3) isolate was considered to be sensitive if absorbance was below the limit of detection.

### 2.4. Carbon Source Assays

One hundred and one isolates, consisting of 87 genera, were evaluated for cellulase, starch degradation, and tannase activity.

#### 2.4.1. Overall Cellulase Activity

Cellulose azure serves as a tool for evaluating the overall activity of cellulases, with most fungi found to produce higher levels of endoglucanase compared to other cellulase enzymes [41]. Endoglucanase, also known as 1,4-β-D-glucan glucanohydrolases (EC. 3.2.1.4), is an enzyme that catalyzes the hydrolysis of internal β−1,4-glycosidic bonds in cellulose [42]. The base medium comprised 5 g of C_4_H_12_N_2_O_6_, 1 g of KH_2_PO_4_, 0.5 g of MgSO_4_*·*7H_2_O, 0.1 g of yeast extract, 0.001 g of CaCl_2_*·*2H_2_O, and 16 g of agar per liter of distilled water. The overlay medium was identical to the base medium but included an additional 1% cellulose azure (Sigma-Aldrich, St. Louis, MO, USA). About 5 mL of the base medium were poured into sterilized glass test tubes, allowed to cool, and then topped with 100 mL of overlay medium. Each test tube was inoculated and observed daily for a period of 10 days. A blue dye movement into the base layer indicated a positive result.

#### 2.4.2. Starch Degradation

Alpha-amylase (EC 3.2.1.1) catalyzes the breakdown of starch granules into smaller maltodextrins via endo-hydrolysis. Subsequently, β-amylase (EC 3.2.1.2) targets alternate α−1,4 links in maltodextrins to yield maltose, while de-branching enzymes act on α−1,6 links to produce linear maltodextrins. Both maltose and linear maltodextrins can be further converted into glucose by α-glucosidase (α-D-glucoside glucohydrolase, EC 3.2.1.20). The starch-based medium contained 1 g of KH_2_PO_4_, 0.5 g of MgSO_4_*·*7H_2_O, 0.1 g of yeast extract, 0.001 g of CaCl_2_*·*2H_2_O, 1% corn starch, and 16 g of agar per liter of distilled water. This medium was sterilized, poured into 60 mm Petri plates, inoculated, and assessed on the 7th day post-inoculation. Gram’s iodine [43] was used to flood the plates, enabling the visualization of clearing zones that indicate a positive result.

#### 2.4.3. Tannic Acid Degradation

Tannase, also known as tannin acylhydrolase (E.C. 3.1.1.20), catalyzes the cleavage of ester and peptide bonds in hydrolyzable tannins and gallic acid esters, producing glucose and gallic acid [44]. The tannase medium comprised 5 g of C_4_H_12_N_2_O_6_, 1 g of KH_2_PO_4_, 0.5 g of MgSO_4_*·*7H_2_O, 0.1 g of yeast extract, 0.001 g of CaCl_2_*·*2H_2_O (from Sigma), and 16 g of agar per liter of distilled water. After sterilization, the medium was cooled to 55 °C before adding filter-sterilized tannic acid. Tannase activity was evaluated using low (0.2 g per liter) and high (0.4 g per liter) concentrations. The medium was poured into 90 mm Petri plates, inoculated, and assessed on the 10th day post-inoculation. A dark zone surrounding the colony indicated a positive tannase activity test [44].

## 3. Results

### 3.1. Fungal Taxa Analyzed

A total of 161 fungal isolates were evaluated for atrazine, 162 fungal isolates were evaluated for cypermethrin and malathion, and 153 fungal isolates were evaluated for mancozeb, encompassing a total of 97 genera (Table 2, Figures S1 and S2, and Data S1). Sixty-eight isolates were from common saltwater marsh plants (*P. australis* and *S. alterniflora*), 43 isolates were from freshwater streams, and 51 isolates were from peatland.

Isolates assayed consisted of four phyla: Ascomycota (139, 85.8%), Basidiomycota (17, 10.5%), Mortierellomycota (3, 1.85%), and Mucoromycota (3, 1.85%). Within the Ascomycota, the five classes with the most representation were Sordariomycetes (61, 37.7%), Dothideomycetes (39, 24.1%), Saccharomycetes (15, 9.3%), Leotiomycetes (11, 6.8%), and Eurotiomycetes (10, 6.2%) (Table 2). The two most represented Basidiomycota classes were Tremellomycetes (11, 6.8%), followed by Microbotryomycetes (4, 2.5%) (Table 2). The class level for both Mortierellomycota and Mucoromycota is *incertae sedis*; therefore, the genus *Mortierella* was represented within the Mortierllomycota, and the genera *Mucor*, *Rhizomucor*, and *Umbelopsis* represented the Mucoromycota (Table 2).

### 3.2. Pesticide Sensitivity Assays

#### 3.2.1. Atrazine and Mancozeb

**Figure 1.**
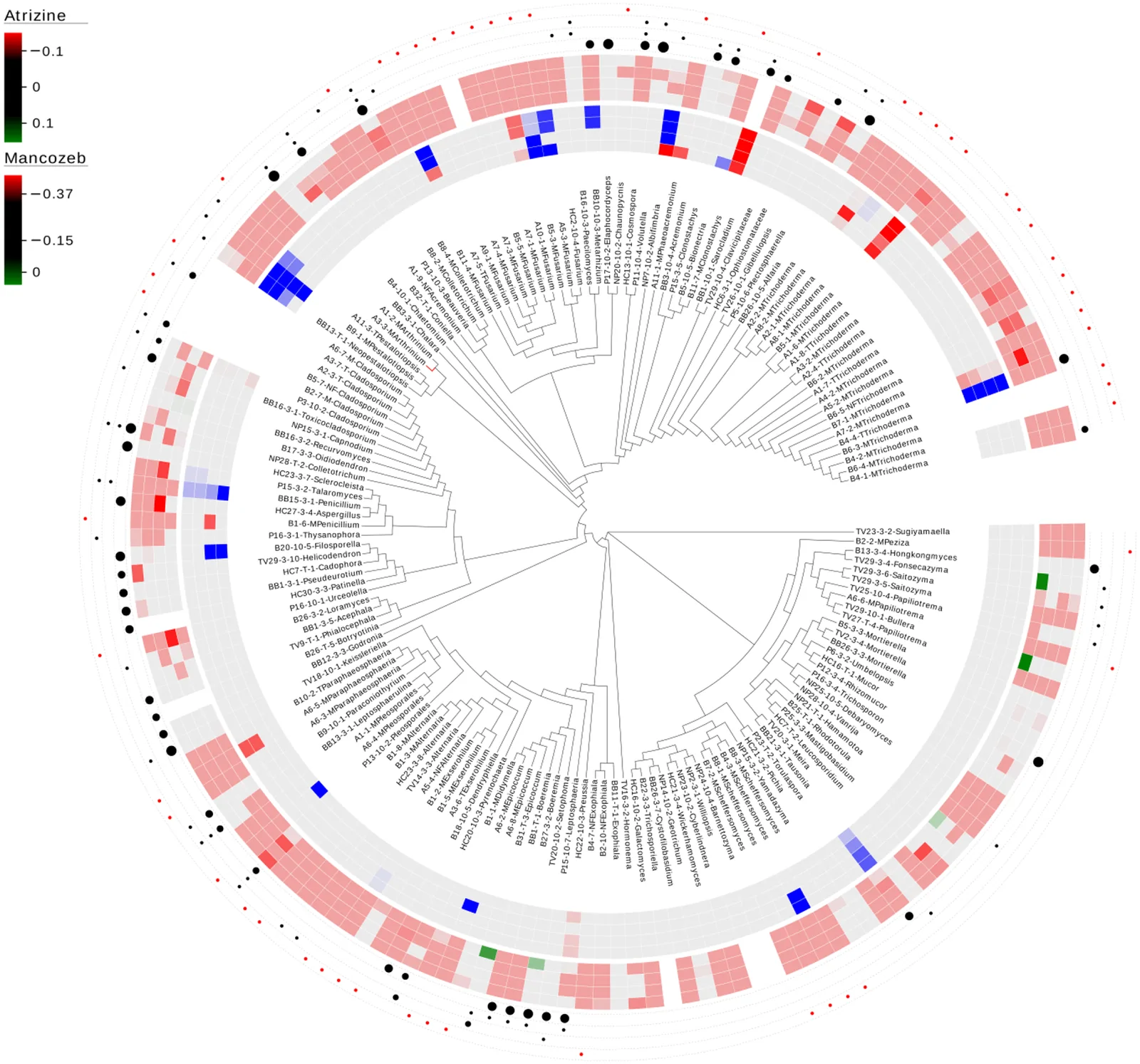
Carbon utilization and sensitivity to environmental levels of atrazine and mancozeb in detritus-inhabiting and saltwater marsh root-associated fungi. The limit of detection was set to 3× standard deviation for each pesticide control concentration. Red indicates negative growth compared to no exposure, whereas blue indicates growth compared to no exposure. The inner circle represents a heat map of the atrazine results at 0.3, 7 ng/L, 50 ng/L, 150 ng/L, and 350 ng/L. The outer heat map represents mancozeb results at 100 μg/L, 500 μg/L, 1.5 mg/L, and 4.0 mg/L. The outer four layers of dots represent the ability to break down cellulose (inner), starch degradation (2nd), tannase activity (3rd), and not tested (outer-represented by a red dot). The size of the cellulose dot represents how fast the isolate showed positive activity, with the largest being the fastest. Dots for starch degradation and tannase activity reflect presence/absence. Blank indicates tested, but no activity detected.

In total, 82% (133/162) of the fungal isolates were within the limit of detection, whereas only three isolates (1.9%) demonstrated lower growth when exposed to the herbicideatrizine over the entire range (3.7 ng/L—350 ng/L) (Figure 1). These three isolates were a *Clonostachys* sp. and two *Trichoderma* species. Three additional isolates (*Fusarium* sp., *Paraphaeosphaeria* sp., and *Preussia* sp.) were consistently sensitive to the two highest concentrations (150 ng/L and 350 ng/L). In contrast, five species (3.1%) had greater consistent growth compared to the control, including *Fusarium* sp., *Penicillium* sp., *Pestalotiopsis* sp., *Tausonia* sp., and *Trichoderma* sp. For mancozeb, 19.6% (30/153) of the fungal isolates were consistently within the limit of detection, whereas 26% (40/153) of the isolates demonstrated inconsistent results. Additionally, 4.6% (7/153) of the isolates showed consistently lower growth compared to the control under the range of 500 ng/L—4.0 mg/L. The majority (49.7% (76/153)) demonstrated consistently lower growth compared to the control across the entire range of 100 μg/L—4.0 mg/L (Figure 1).

#### 3.2.2. Cypermethrin and Malathion

**Figure 2.**
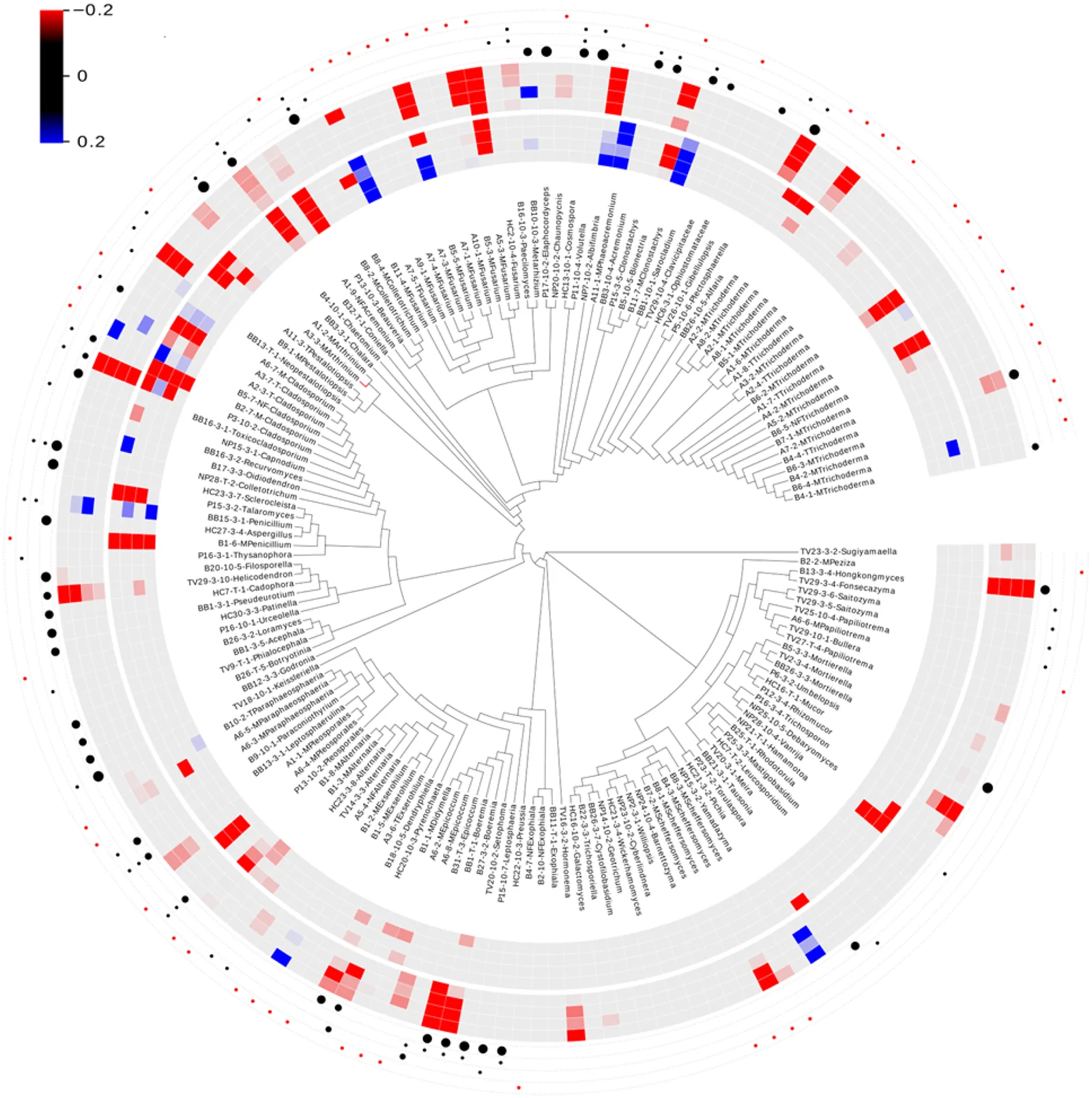
Carbon utilization and sensitivity to environmental levels of cypermethrin and malathion in detritus-inhabiting and saltwater marsh root-associated fungi. Sensitivity of fungal isolates at the 0.15 cut-off level. Red indicates negative growth compared to no exposure, whereas blue indicates growth compared to no exposure. The inner circle represents a heat map of the cypermethrin results at 0.5 μg/L, 1 μg/L, 3 μg/L, and 6 μg/L. The outer heat map represents Malathion results at 1 μg/L, 10 μg/L, 20 μg/L, and 40 μg/L. The outer four layers of dots represent the ability to break down cellulose (inner), starch degradation (2nd), tannase activity (3rd), and not tested (outer-represented by a red dot). The size of the cellulose dot represents how fast the isolate showed positive activity, with the largest being the fastest. Dots for starch degradation and tannase activity reflect presence/absence. Blank indicates tested, but no activity detected.

For cypermethrin, 72.8% (118/162) of the fungal isolates were within the limit of detection, whereas only five isolates (*Beauveria* sp., *Cladosporium* sp., *Coniella lustricola*, *Penicillium* sp., *Trichoderma* sp.) demonstrated consistently lower growth when exposed to the insecticide over the entire range (0.5 μg/L—6 μg/L) (Figure 2). An additional five isolates (3.1%) demonstrated lower growth when exposed to the range of 1 μg/L—6 μg/L. Only 1.9% (3/162) demonstrated consistent growth over the entire range and consisted of *Clonostachys* sp., *Fusarium* sp., and *Phaeoacremonium* sp. The remaining 31 isolates displayed inconsistent results across the entire range. For malathion, 73.5% (119/162) of the fungal isolates were within the limit of detection. Nine fungal isolates (5.6%) demonstrated lower growth over the entire range of 1 μg/L—40 μg/L, which consisted of *Albifimbria* sp., *Alfaria* sp., *Boeremia* sp., *Cladosporium* sp., *Coniella lustricola*, *Fusarium* sp., *Helicodendron* sp., *Hongkongmyces snookiorum*, and *Trichoderma* sp. An additional eight isolates displayed reduced growth under the concentration range of 10 μg/L—40 μg/L, while *Pichia* sp. displayed increased growth under the same concentration range (Figure 2).

#### 3.2.3. Species-Specific Function and Comparison to Sensitivity Assays

One hundred and one isolates, consisting of 87 genera, were evaluated for cellulase, starch degradation, and tannase activity (Figures 1 and 2). Forty-two (41.6%) tested positive for overall cellulase activity across 35 genera, whereas only 28.7% of the isolates (29/101) demonstrated starch degradation across 24 genera. Tannase activity was demonstrated by 31 isolates (30.7%) across 22 genera. Only six isolates tested positive for all three and consisted of two *Colletotrichum* spp., *Coniella lustricola*, *Cosmospora* sp., *Leptosphaerulina* sp., and *Pleosporales* sp. (Figures 1 and 2, Data S1).

## 4. Discussion

In this study, we evaluated the effects of four pesticides on 153–162 fungal isolates, representing 97 genera, from diverse aquatic environments. Notably, this study stands as one of the few comprehensive surveys investigating the impacts of pesticides on such a wide array of fungal genera. Previous research has demonstrated that some fungi possess the ability to break down pesticides [45,46], whereas others indicate the detrimental effects of pesticides on fungi [47]. We observed that a majority of the fungal isolates exhibited tolerance to atrazine, cypermethrin, and malathion. While it was expected that many fungi would show reduced growth when exposed to the fungicide mancozeb, the low solubility of this pesticide led to higher variation within the data compared to the other three pesticides, making it challenging to draw definitive conclusions. Consequently, further refinement of this method is necessary to obtain more conclusive results regarding the sensitivity of these isolates to mancozeb. Crucially, our findings suggest that the breakdown of cellulose, starch, and tannins by the fungal community is unlikely to decrease upon exposure to these pesticides at the examined environmental levels. This inference is drawn from the observed functional redundancy across different isolates and genera (see Figures 1 and 2). Below, we provide a detailed review of the species in this study that consistently exhibited negative or positive growth when exposed to atrazine, cypermethrin, and malathion. Additionally, we discuss the sensitivity of fungi to pesticides in relation to community functional redundancy.

### 4.1. Pesticide Sensitivity

Atrazine, an herbicide that inhibits electron transport in photosystem II in plants, is one of the most widely used herbicides. Research has suggested that fungi can remove the side chains of atrazine via N-dealkylation [45]. In this study, *Clonostachys* sp., *Trichoderma* spp., *Fusarium* sp., *Paraphaeosphaeria* sp., and *Preussia* sp. were consistently sensitive to all or consistently sensitive to the two highest concentrations of atrazine (Figure 1). In contrast, a different *Fusarium* sp., *Penicillium* sp., *Pestalotiopsis* sp., *Tausonia* sp., and *Trichoderma* sp. had consistent growth when exposed, suggesting that these species were tolerant or able to break down atrazine. Similarly, research investigating the effects of atrazine on the microbial population of marine coastal surface water showed that exposure to atrazine altered both the overall number and diversity of bacteria and fungi [48]. These authors also indicated that genera mainly isolated from atrazine-treated seawater were *Aspergillus*, *Candida*, *Fusarium*, and *Saccharomyces*. For this study, the *Aspergillus* sp. was within the limit of detection, meaning it appeared tolerant but did not grow greater than the control. We assayed eleven *Fusarium* spp. with only one species sensitive to atrazine; three species demonstrated positive growth under at least one concentration, and the remaining seven species were all within the limit of detection. Importantly, we did not detect any phylogenetic signature or clustering for atrazine sensitivity, but we do note that two *Pestalotiopsis* spp. and *Neopestalotiopsis* demonstrated positive growth at the two highest concentrations (Figure 1). Consequently, it would be beneficial to evaluate other species within these genera for their potential to break down atrazine. Understanding the sensitivity and potential degradation mechanisms of various microbial species toward atrazine is crucial for developing effective strategies for herbicide management and environmental protection.

Cypermethrin, a synthetic pyrethroid, is an insecticide that works by inducing neuronal hyperexcitation, leading to paralysis and death. The reported half-life depends on the environment, ranging from 4 to 65 days in soils [49] and extending up to 94 to 1103 days in other environments [50]. Consequently, environmental accumulation can occur. Research has identified several bacterial species capable of degrading cypermethrin, including *Bacillus subtilis* [49] and *Pseudomonas aeruginosa* [51]. Similarly, another study showed that exposure to cypermethrin increased the growth of organotrophic bacteria and actinomycetes but was detrimental to overall fungal growth [52]. The same studies identified that cypermethrin was tolerated by a *Penicillium* sp. and a *Trichocladium* sp. In our study, *Beauveria* sp., *Cladosporium* sp., *Coniella lustricola*, one *Penicillium* sp., and a *Trichoderma* sp. demonstrated consistently lower growth when exposed to cypermethrin, whereas *Clonostachys* sp., *Fusarium* sp., and a *Phaeoacremonium* sp. exhibited consistent growth when exposed to cypermethrin (Figure 2). Similar to the previous study, another *Penicillium* sp. that we assayed showed positive growth, but it was inconsistent across all assay concentrations. Importantly, we did not find any phylogenetic signal associated with cypermethrin sensitivity or tolerance across the diverse genera we examined. Understanding the response of microbial communities to cypermethrin exposure is crucial for assessing the environmental impact of this widely used insecticide and for developing effective mitigation strategies.

Malathion, an organophosphate insecticide, functions by inactivating acetylcholinesterase, leading to hyper-excitation of the nervous system. Research has demonstrated that it is degraded by microbes through soluble carboxylesterase enzymes [53]. Bacterial strains from various genera, including *Pseudomonas* [53], *Flavobacterium*, and *Xanthomonas* [54], have been found capable of degrading malathion. Additionally, isolates within the fungal genera *Aspergillus*, *Penicillium*, and *Trichoderma* have been reported to break down malathion [53,55]. It has also been reported that bacteria degrade malathion more rapidly compared to fungi [55]. In our study, *Albifimbria* sp., *Alfaria* sp., *Boeremia* sp., *Cladosporium* sp., *Coniella lustricola*, *Fusarium* sp., *Helicodendron* sp., *Hongkongmyces snookiorum*, and a *Trichoderma* sp. were consistently sensitive to malathion (Figure 2). Interestingly, both assayed *Boeremia* spp. appeared to be sensitive, although no overall phylogenetic pattern for sensitivity was evident. For example, of the 21 *Trichoderma* isolates assayed, only one *Trichoderma* sp. was consistently sensitive, one isolate was sensitive at concentrations ranging from 10 μg/L to 40 μg/L, and the remaining isolates were all within the limit of detection. One isolate, a *Pichia* sp., in our study, exhibited growth under malathion exposure. Previous research has shown that *Pichia kluyveri* could break down the organochlorine pesticide dichlorodiphenyltrichloroethane [56], suggesting that other members of this genus should be assayed for their bioremediation potential. Understanding the sensitivity of various microbial species to malathion is essential for assessing the risks associated with its use and for implementing appropriate environmental management strategies.

### 4.2. Functional Redundancy

Fungal-mediated environmental processes hinge on a diverse community of species breaking down various carbon resources in the environment. Therefore, establishing a connection between environmental function and individual species is crucial for comprehending fungal-driven processes. Building on prior investigations [57], this study indicates that closely related fungi might degrade similar substrates. However, it also uncovers evidence that closely related fungi exhibit variations in their efficiency in degrading a given substrate (Figures 1 and 2). Furthermore, [57] observed that eight *Aspergillus* spp. possessed comparable genomic capabilities for degrading plant biomass. Nonetheless, each species displayed distinct overall activities and utilized specific enzymes for degrading plant biomass. In our broad survey, we find that isolates from many genera are functionally redundant in cellulase and tannase activity and starch degradation. More importantly, there were functionally redundant isolates tolerant to atrazine, cypermethrin, and malathion. This suggests that tolerant species could make up for losses in environmental function if those that are sensitive to these pesticides were to be removed. Ultimately, additional functional studies need to be completed to determine the extent to which these pesticides may limit fungal-driven processes. In addition, particular interest should focus on understanding the impact of pesticides on mutualist fungi and their hosts since these partnerships would lack the diversity and functional redundances seen in generalized saprobic or general root-associated fungi.

## 5. Conclusions

This research identifies functional redundancy across and within genera and provides evidence that the breakdown of cellulose, starch, and tannins by the fungal community is unlikely to decrease when exposed to atrazine, cypermethrin, and malathion; however, further refinement of this method is necessary to obtain more conclusive results regarding the sensitivity of these isolates to mancozeb. Through a broad survey encompassing up to 97 fungal genera and four levels of pesticide exposure, we found no phylogenetic signal for sensitivity or tolerance to these three pesticides. This appears to agree with findings by [58], that concluded the sensitivity to pesticides can vary between *Beauveria* spp. and strains of the sample species. However, continued sampling and tolerance assays will be needed to determine the extent of pesticide sensitivity across the fungal kingdom. With the continued increase in pesticide use leading to higher levels of environmental contamination, it is increasingly crucial to understand their effects on non-target microbes and assess their potential impact on vital fungal-driven environmental processes.

## Supplementary Material

Data S1 Molecular and assay results-updated

Figures S1, S2

## Figures and Tables

**Table 1.** Sample site data for obtaining fungal isolates for pesticide evaluation.

Sampling Locations	State	Collection	Type	Fungal Habitat
Black Moshannon State Park (42.819°/−88.407)^1^	Pennsylvania	2014	fen	fine detritus
Tannersville Cranberry bog (41.037/−75.264)	Pennsylvania	2016	acid-poor fen	fine detritus
Pepper Run (41.051/−77.331)	Pennsylvania	2014	first-order stream	fine detritus
Nescopeck State Park (41.093/−78.059)	Pennsylvania	2016	first-order stream	fine detritus
Beulah bog (42.819/−88.407)	Wisconsin	2016	kettle bog	fine detritus
Honey Creek Natural Area (42.727/−88.269)	Wisconsin	2016	first-order stream	fine detritus
Republic (36.792/−76.289)	Virginia	2019	saltwater marsh	root-associated
Bell Mills (36.721/−76.253)	Virginia	2019	saltwater marsh	root-associated
Deep Creek Lock (36.746/−76.343)	Virginia	2019	saltwater marsh	root-associated

^1^ Data represents latitude and longitude values.

**Table 2.** Species of aquatic detritus-inhabiting and aquatic root-associated fungi assayed for sensitivity to atrazine, cypermethrin, malathion, and mancozeb.

Phylum/Class	Genus	Species or Final Determination	Number of Isolates Per Taxonomic Level	Isolation^1^
Ascomycota				
Dothideomycetes				
	*Alternaria*	*alternata*	2	D
		sp.	4	RA/D
	*Boeremia*	*exigua*	1	D
		sp.	1	D
	*Capnodium*	sp.	1	D
	*Cladosporium*	*cladosporioides*	1	D
		sp.	5	RA
	*Dendryphiella*	sp.	1	D
	*Didymella*	sp.	1	RA
		Didymellaceae sp.	2	RA
	*Epicoccum*	*nigrum*	1	D
	*Exserohilum*	*rostratum*	3	RA
	*Hongkongmyces*	*snookiorum*	1	D
	*Hormonema*	sp.	1	D
	*Keissleriella*	*yonaguniensis*	1	D
	*Leptosphaeria*	sp.	1	D
	*Leptosphaerulina*	*chartarum*	1	D
	*Paraconiothyrium*	*fuckelii*	1	D
	*Paraphaeosphaeria*	sp.	3	RA
	*Preussia*	*flanaganii*	1	D
	*Pyrenochaeta*	*unguis-hominis*	1	D
	*Recurvomyces*	*mirabilis*	1	D
	*Tausonia*	*pullulans*	1	D
	*Toxicocladosporium*	*ficiniae*	1	D
		Unknown	3	RA/D
Eurotiomycetes				
	*Aspergillus*	*fumigatus*	1	D
	*Exophiala*	*spartinae*	3	RA
	*Paecilomyces*	sp.	1	D
	*Penicillium*	sp.	2	RA/D
	*Sclerocleista*	*ornata*	1	D
	*Talaromyces*	sp.	1	D
	*Thysanophora*	*penicilliodes*	1	D
Incertae sedis				
	*Filosporella*	sp.	1	D
	*Pseudeurotium*	sp.	1	D
	*Setophoma*	*vernoniae*	1	D
Leotiomycetes				
	*Acephala*	sp.	1	D
	*Botryotinia*	*fuckeliana*	1	D
	*Cadophora*	*luteo-olivacea*	1	D
	*Godronia*	*cassandrae*	1	D
	*Helicodendron*	*triglitziense*	1	D
	*Loramyces*	*macrosporus*	1	D
	*Patinella*	*hyalophaea*	1	D
	*Phialocephala*	*fortinii*	1	D
	*Trichosporiella*	sp.	1	D
	*Urceolella*	*carestiana*	1	D
Pezizomycetes				
	*Peziza*	sp.	1	RA
Saccharomycetes				
	*Barnettozyma*	*californica*	1	D
	*Cyberlindnera*	*saturnus*	1	D
	*Debaryomyces*	*hansenii*	1	D
	*Galactomyces*	*candidum*	1	D
	*Geotrichum*	*restrictum*	1	D
	*Pichia*	*kudriavzevii*	1	D
	*Scheffersomyces*	*spartinae*	4	RA
	*Sugiyamaella*	*paludigena*	1	D
	*Torulaspora*	*delbrueckii*	1	D
	*Wickerhamomyces*	*anomalus*	1	D
	*Williopsis*	sp.	1	D
	*Yamadazyma*	*mexicana*	1	D
Sordariomycetes				
	*Acremonium*	*rutilum*	2	RA/D
	*Albifimbria*	*viridis*	1	D
	*Alfaria*	sp.	1	D
	*Arthrinium*	*arundinis*	2	RA
	*Beauveria*	*brongniartii*	1	D
	*Bionectria*	sp.	1	D
	*Chaetomium*	sp.	1	D
	*Chalara*	*vaccinii*	1	D
	*Chaunopycnis*	sp.	1	D
		Clavicipitaceae sp.	1	D
	*Clonostachys*	sp.	2	RA/D
	*Colletotrichum*	sp.	3	RA/D
	*Coniella*	*lustricola*	1	D
	*Cosmospora*	sp.	1	D
	*Elaphocordyceps*	sp.	1	D
	*Fusarium*	*equiseti*	1	D
		sp.	7	RA
		*sporotrichioides*	3	RA
	*Metarhizium*	*anisopliae*	1	D
	*Neopestalotiopsis*	sp.	1	D
		Ophiostomataceae sp.	1	D
	*Pestalotiopsis*	sp.	2	RA
	*Phaeoacremonium*	sp.	1	RA
	*Plectosphaerella*	*cucumerina*	1	D
	*Sarocladium*	sp.	1	D
	*Trichoderma*	atroviride	2	RA
		sp.	19	RA/D
	*Volutella*	sp.	1	D
Basidiomycota				
Exobasidiomycetes				
	*Meira*	sp.		D
Microbotryomycetes				
	*Hamamotoa*	sp.	1	D
	*Leucosporidium*	sp.	1	D
	*Mastigobasidium*	*intermedium*	1	D
	*Rhodotorula*	*bogoriensis*	1	D
Tremellomycetes				
	*Bullera*	*alba*	1	D
	*Cystofilobasidium*	*macerans*	1	D
	*Fonsecazyma*	*betulae*	1	D
	*Gibellulopsis*	*nigrescens*	1	D
	*Papiliotrema*	*flavescens*	1	D
		*pseudoalba*	1	RA
		sp.	1	D
	*Saitozyma*	*podzolica*	2	D
	*Trichosporon*	*moniliiforme*	1	D
	*Vanrija*	*musci*	1	D
Mortierellomycota				
Incertae sedis				
	*Mortierella*	*macrocystis*	1	D
		*sossauensis*	1	D
		Mortierellales sp.	1	D
Mucoromycota				
Incertae sedis				
	*Mucor*	sp.	1	D
	*Rhizomucor*	*variabilis*	1	D
	*Umbelopsis*	*ramanniana*	1	D

^1^ D = fine detritus, RA = root associated.

**Table 3.** Pesticide information and assay concentrations.

Chemical	Water Solubility	Environmental Levels	Assay Concentrations ^−L^			
			1	2	3	4
Mancozeb	6–20 mg/L	1–4 mg/L^1^	100 μg	500 μg	1.5 mg	4 mg
Malathion	130 mg/L	0.3 to 38 μg/L	1 μg	10 μg	20 μg	40 μg
Cypermethrin	9 μg/L	6.25 μg/L	0.5 μg	1 μg	3 μg	6 μg
Atrazine	33 μg/mL	3.7 to 350 ng/L	3.7 ng	50 ng	150 ng	350 ng

^1^ Level of toxicity to daphnia and algae.

## Data Availability

All pesticide assay results, carbon source results, and molecular results are provided in Supplemental Data S1.
