# Supplementary material for: Evaluation of Pesticides on Detritus-Inhabiting and Root-Associated Fungi in Aquatic Habitats and Potential Implications
Source: Diversity (Basel). Author manuscript; Available in PMC 2026 Aug 25. (PMC13502160; doi:10.3390/d16050255)
Supplement: Supplementary file 1 [file NIHMS2150929-supplement-Figures_S1__S2.pdf]

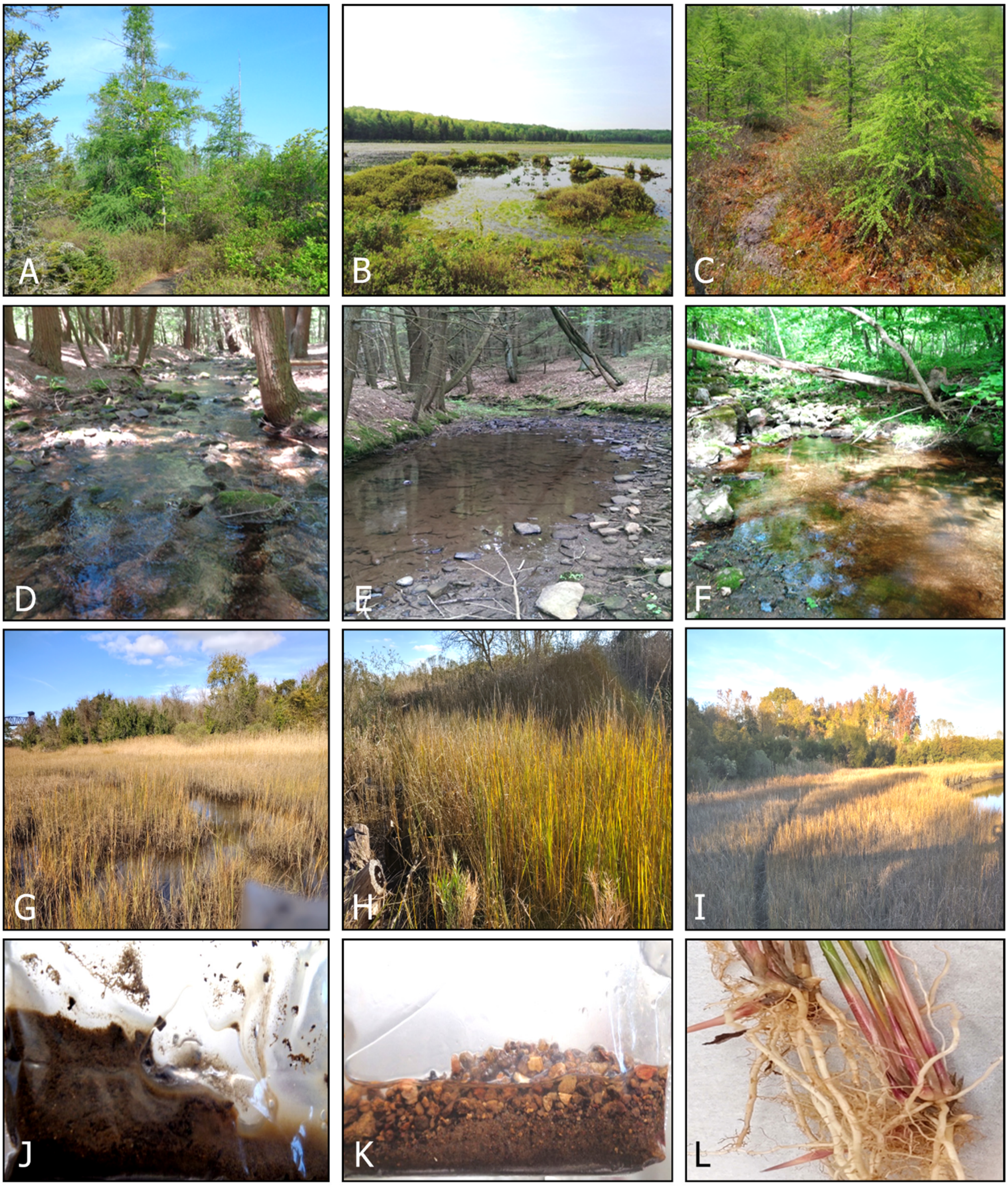

Figure S1. Sampling sites and samples. A) Tannersville Cranberry bog B) Black Moshannon State Park C) Beulah bog D) Nescopeck State Park E) Pepper Run F) Honey Creek Natural Area G) Republic H) Bell Mills I) Deep Creek Lock J) detritus sample K) stream bed sample L) roots.

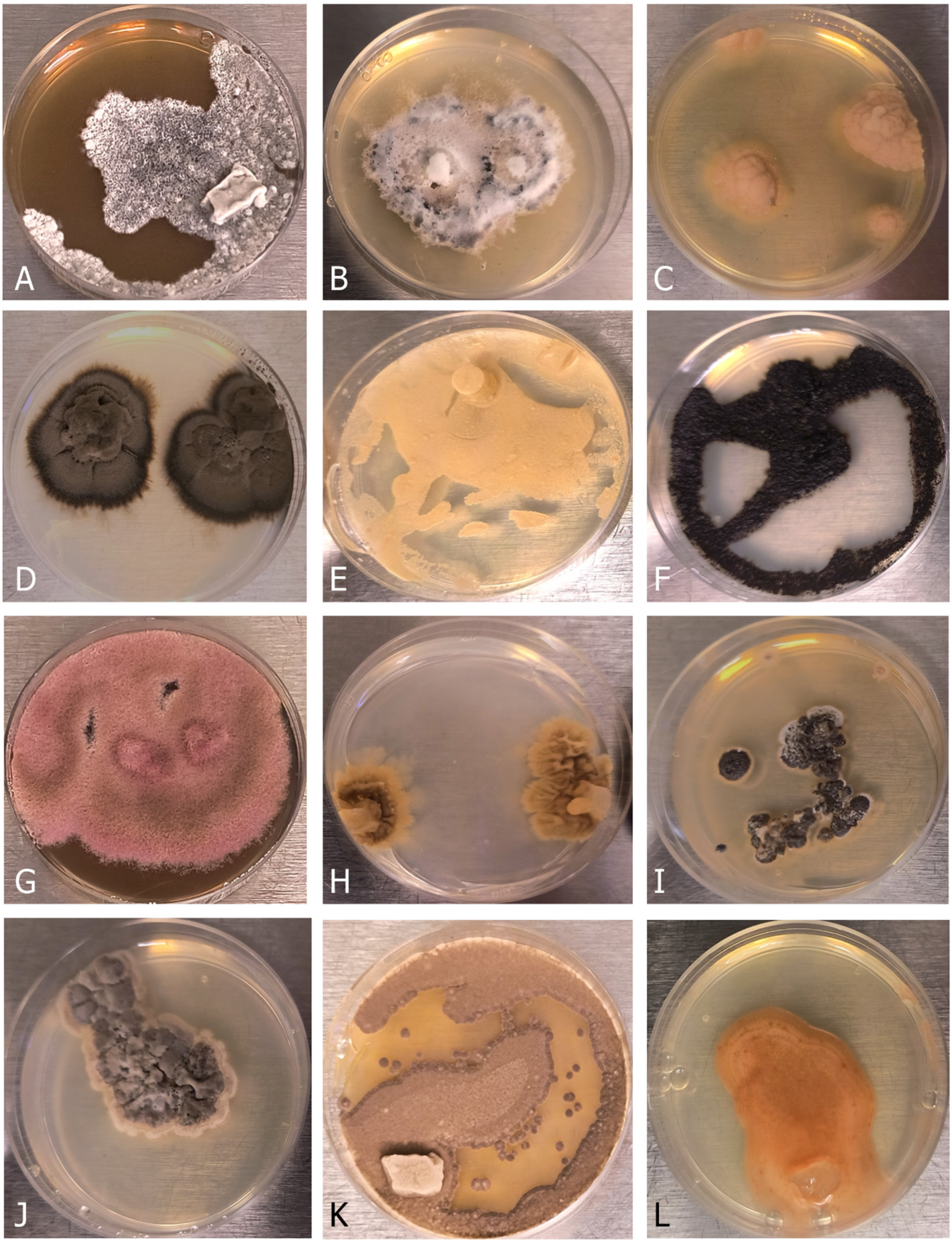

Figure S2. A representation of detritus-inhabiting and saltwater marsh root-associated fungi. A) Acremonium sp. B) Alternaria sp. C) Aureobasidium sp. D) Cladosporium sp. E) Cryptococcus sp. F) Exophiala sp. G) Fusarium sp. H) Leotiomycetes sp. I) Mycosphaerella sp. J) Paraphaeosphaeria sp. K) Penicillium sp. and L) Rhodotorula sp.
